# Association of Simultaneous vs Delayed Resection of Liver Metastasis With Complications and Survival Among Adults With Colorectal Cancer

**DOI:** 10.1001/jamanetworkopen.2022.31956

**Published:** 2022-09-19

**Authors:** Yibin Wu, Anrong Mao, Huipeng Wang, Guojiu Fang, Jiamin Zhou, Xigan He, Sanjun Cai, Lu Wang

**Affiliations:** 1 Department of Hepatic Surgery, Fudan University Shanghai Cancer Center, Shanghai Medical College, Fudan University, Shanghai, China; 2 Department of General Surgery, Fifth People's Hospital of Shanghai, Fudan University, Shanghai, China; 3 Department of General Surgery, Shanghai Fengxian District Central Hospital, Shanghai, China; 4 Department of Colorectal Surgery, Fudan University Shanghai Cancer Center, Shanghai Medical College, Fudan University, Shanghai, China

## Abstract

**Importance:**

Simultaneous or delayed resection of synchronous liver metastasis (SLM) with primary colorectal cancer (CRC) remains a controversial topic.

**Objective:**

To investigate the outcomes of simultaneous vs delayed resection in patients with resectable SLM.

**Design, Setting, and Participants:**

This comparative effectiveness research study included 1569 patients with resectable SLM who underwent curative-intent liver resection at 3 independent centers in China between January 1, 2000, to December 31, 2019. A 1:1 propensity score matching was performed. Follow-up was completed on August 31, 2021, and the data were analyzed from April 1 to 30, 2022.

**Main Outcomes and Measures:**

Primary outcome was the percentage of patients with at least 1 major complication within 60 days after surgery. Secondary outcomes were intraoperative and postoperative complications, overall survival (OS), and cancer-specific survival (CSS) rates.

**Results:**

Among the 1569 patients included, 1057 (67.4%) underwent delayed resection (719 men [68.0%] with a mean [SD] age of 57.4 [11.2] years), and 512 patients (310 men [60.5%] with a mean [SD] age of 57.1 [10.5] years) underwent simultaneous resection. Matching yielded 495 pairs of patients underwent simultaneous resection. The percentage of major perioperative complications did not differ between the simultaneous and delayed resection groups (34.1% vs 30.0%; *P* = .89). The OS rates were 65.2% at 3 years, 47.1% at 5 years, and 38.0% at 8 years for the delayed resection group and 78.0% at 3 years, 65.4% at 5 years, and 63.1% at 8 years for the simultaneous resection group (hazard ratio [HR], 1.42; 95% CI, 1.10-1.85, *P* = .003). The CSS rates were 68.3% at 3 years, 48.5% at 5 years, and 37.1% at 8 years for the delayed resection group and 79.2% at 3 years, 67.2% at 5 years, and 65.9% at 8 years for the simultaneous resection group (HR, 1.45; 95% CI, 1.14-1.98; *P* = .004). On subgroup analysis comparing the 2 strategies according to the *KRAS* sequence variation status, the OS rates (HR, 1.61; 95% CI, 1.45-2.18; *P* < .001) and CSS rates (HR, 1.62; 95 CI, 1.40-1.87; *P* = .003]) in the simultaneous resection group were significantly better than those in the delayed resection group in patients with *KRAS* wild-type tumors.

**Conclusions and Relevance:**

Results of this study suggest that complication rates did not differ when CRC and SLM were resected simultaneously and that the survival benefits of simultaneous resection were restricted to patients with *KRAS* wild-type tumors. Integrating molecular features into the treatment decision is a basis for accurate, individualized treatments.

## Key Points

**Question:**

What is the association of simultaneous vs delayed resection of liver metastases with major complications and survival in adults with colorectal cancer with or without *KRAS* sequence variation?

**Findings:**

In this comparative effectiveness research study of 1569 patients with resectable synchronous liver metastases, simultaneous resection was associated with longer survival rates than delayed resection in patients with *KRAS* wild-type tumors but not in patients with *KRAS* sequence variation.

**Meaning:**

Findings of this study suggest that integrating molecular features in the choice of treatment provides a theoretical basis for accurate, individualized treatments.

## Teaser

This comparative effectiveness research examines overall survival and cancer-specific survival in patients with or without* KRAS* sequence variation who received treatment for synchronous liver metastases.

## Introduction

Up to one-fifth of patients with colorectal cancer (CRC) present with synchronous liver metastasis (SLM).^1,2^ Surgical management to remove the primary tumor and liver metastatic burden is advocated by current guidelines. Surgery for CRC with resectable SLM can take several forms such as (1) the classic approach, involving initial colorectal resection, interval chemotherapy, and liver resection or a liver-first approach with removal of the colorectal tumor as the final procedure, and (2) simultaneous removal of the liver and bowel tumors with or without neoadjuvant or adjuvant chemotherapy.^3^ However, for patients with CRC and SLM, the timing of colorectal and liver surgery remains controversial.

Reported comparisons of the 2 strategies have been somewhat inconsistent, and findings have been reported in 5 reviews and meta-analyses. One study found that the postoperative morbidity rate of patients who underwent simultaneous resection was substantially lower,^4^ whereas other studies found that the 2 strategies may not differ in terms of postoperative complications and survival outcomes.^5,6,7^ To date, only 1 randomized clinical trial that compared outcomes among the simultaneous and the classic approaches has been published: the METASYNC (Simultaneous Vs Delayed Resection for Initially Resectable Synchronous Colorectal Liver Metastasis) trial.^8^ The trial demonstrated no difference between the 2 approaches in postoperative complications within 60 days of surgery. The simultaneous resection group had superior 2-year overall survival (OS) and disease-free survival compared with the delayed resection group.^8^ Despite these findings, drawing robust conclusions from this trial is difficult because of the limited number of patients and substantial heterogeneity in tumor characteristics. Moreover, it lacked power to assert that simultaneous resection was recommended in all situations, particularly in certain patients who harbored specific sequence variations.

Kirsten rat sarcoma viral oncogene homologue (*KRAS;* OMIM: 114500) is the best-known oncogene with the highest rate of sequence variation among all cancers and has exhibited a role in revolutionizing cancer treatment strategies.^9,10,11^ Previous studies have highlighted the benefits of anatomical resections in liver and lung mastectomy of CRC with *KRAS* sequence variation.^12,13^ However, the association of integrating *KRAS* data with the benefit of simultaneous vs delayed resection of liver metastases is largely unknown.

We aimed to investigate the outcomes of simultaneous vs delayed resection in patients with resectable SLM using a multicentric propensity cohort. The primary outcome was the percentage of patients with major complications occurring at a surgical site. Secondary outcomes were the 10-year OS and cancer-specific survival (CSS) rates. Furthermore, we sought to stratify the benefit of simultaneous vs delayed resection of liver metastases according to the *KRAS* sequence variation status.

## Methods

### Study Population

The study cohort comprised patients who underwent curative-intent surgery for colorectal liver metastasis (CRLM) between January 1, 2000, and December 31, 2019, and who had genetic data retrospectively identified from the patient records of 3 independent centers in China (Fudan University Shanghai Cancer Center, Fifth People’s Hospital of Shanghai Fudan University, and Shanghai Fengxian Central Hospital). The institutional review boards of all participating institutions approved this study and waived the informed consent requirement because of the retrospective nature of the study. The report of this study followed the International Society for Pharmacoeconomics and Outcomes Research (ISPOR) reporting guideline.

Standard demographic and clinicopathological data were collected on each patient, including sex, age, tumor characteristics, operative details, perioperative status, type and time of chemotherapy, molecular features, and date of last follow-up or date of death. Primary tumor characteristics, including tumor location, T (tumor) stage (per the TNM classification by the American Joint Committee on Cancer), nodal status, and tumor differentiation, were recorded. Size, number, and distribution of the liver metastases, as defined by the resection specimen, were also recorded. The largest lesion was used as the index lesion in patients with multiple tumors. Size of 5 cm and 10 lesions were identified as the cutoff.^14,15^

Patients who met all of the following criteria were eligible for inclusion in this study: (1) diagnosis of initially resectable CRC and SLM with pathological confirmation, (2) no other malignant tumors, (3) no multiple colorectal tumors, (4) no extrahepatic metastasis, (5) no incomplete clinical data or insufficient follow-up, and (6) curative intent. Patients with death from unknown cases were excluded from the analysis of CSS. The flowchart of patients is shown in the eFigure in the Supplement.

### Surgery

Synchronous liver metastasis was defined as liver metastases at or before diagnosis of the primary CRC.^14,16^ Before surgery, all tumors were retrospectively assessed by expert groups who reviewed all pretreatment computed tomography scans or magnetic resonance imaging examinations to decide the treatment strategy. Patients with disease considered to be resectable were assigned to undergo liver resection with curative intent, with the aim of achieving complete resection while preserving as much normal, functional liver parenchyma (with adequate vascular inflow, outflow, and biliary drainage) as possible.

Major hepatectomy was defined as resection of 3 or more liver segments^17^ and was performed if the future remaining liver volume was estimated to be sufficient to prevent postoperative liver failure. The future remaining liver is usually calculated as a ratio of the remnant liver volume to the total functional liver volume. In major liver resection, it is generally accepted that this ratio needs to be at least 30% to 40% to fit the hepatic metabolic demands of the recipient. The techniques implemented were independently determined by each surgeon, such as open or laparoscopic approach, manual suture or mechanical anastomosis, tools for hepatic parenchyma transection and hemostasis, and additional radiofrequency ablation if necessary. Perioperative adjuvant chemotherapy was administered according to the protocols in each center.

### *KRAS* Sequence Variation Profiling

As previously described, the extracted DNA was evaluated for the presence of the most common sequence variations of the *KRAS* gene (codons 12 and 13).^18^ These regions of interest were amplified using polymerase chain reaction, and the reaction product underwent agarose gel electrophoresis against known positive (*KRAS*) and negative (*GAPDH* gene) controls to assess the presence and size of the amplified product. Either primary or metastatic tissue was used for the measurements given that a high concordance of the *KRAS* sequence variation status between primary and corresponding metastases has been reported.

### Study Outcomes

The primary outcome was the percentage of patients with at least 1 major complication within 60 days after surgery for CRC and/or SLM. Major complications were validated for each patient by the data validation committee and included the following: (1) digestive complications: intraoperative or postoperative bleeding requiring blood transfusion or reoperation, necrosis of the colon or small bowel, peritonitis, intraabdominal abscess, anastomosis fistula requiring percutaneous drainage or reoperation, and bowel obstruction requiring reoperation; (2) hepatic complications: intraoperative or postoperative bleeding requiring at least 1 unit of blood transfusion or reoperation, bile leakage requiring percutaneous drainage or reoperation, bile duct stenosis requiring endoscopic or percutaneous stenting or reoperation, subphrenic abscess requiring percutaneous drainage or reoperation, and severe liver failure (defined by factor V <30%) on postoperative day 3; and (3) general complications: perioperative death, pulmonary embolism, wound abscess requiring reoperation, pulmonary infection requiring antibiotics and or pleural drainage, severe sepsis (defined by the association of an infection and at least 2 parameters of systemic inflammatory response syndrome) or septic shock, and acute kidney insufficiency. The Clavien-Dindo classification^19^ was added to the protocol to define major complications.

Overall survival was considered to be the time between the day of diagnosis and the day of death or last follow-up. Cancer-specific survival was calculated as the time from diagnosis to last follow-up or death attributed to CRLM. Follow-up was completed on August 31, 2021.

### Statistical Analysis

Patients in the simultaneous resection and delayed resection groups were compared using an unpaired, 2-tailed *t* test for continuous data and the χ^2^ test for categorical data. Survival curves were constructed with the Kaplan-Meier method and compared using the Cox proportional hazards regression model. Subgroup analyses were done according to the *KRAS* sequence variation status. Propensity score matching was used to control selection bias and to create demographically and clinically comparable cohorts. The propensity score was estimated using the nonparsimonious multivariate logistic regression according to sex, tumor sidedness, N (nodal) stage, and anticipated hepatectomy. Patients were matched at a 1:1 ratio using the caliper matching method within 0.20 of the SD of the logit of the propensity score.

Statistical analysis was performed with SPSS software, version 22.0 (IBM Corp), and visualization was created with GraphPad Prism, version 9.0.0 (GraphPad Software). Differences of 2-sided *P* < .05 were considered to be statistically significant. Data were analyzed from April 1 to 30, 2022.

## Results

Among 1569 eligible patients, 1057 (67.4%; 719 men [68.0%] and 338 women [32.0%]) with a mean (SD) age of 57.4 (11.2) years underwent delayed resection, and 512 patients (32.6%; 310 men [60.5%] and 202 women [39.5%]) with a mean (SD) age of 57.1 [10.5] years underwent simultaneous resection. Patient characteristics by treatment group are provided in Table 1. A higher proportion of patients in the simultaneous resection group compared with the delayed resection group were women (39.5% vs 32.0%; *P* = .004), had right-sided tumors (43.2% vs 20.3%; *P* < .001), had an earlier nodal stage at presentation (N2: 26.1% vs 32.8%; *P* = .02), and were more often treated with previous radiotherapy or chemotherapy (47.1% vs 32.3%; *P* < .001) and minor hepatectomy (80.7% vs 74.2%; *P* = .004). The percentages of demographic characteristics, such as age, sex, body mass index, tumor stage, tumor differentiation, metastatic sites, *KRAS* or *BRAF* sequence variation status, and postoperative chemotherapy, did not differ between the 2 groups.

**Table 1.** Baseline Characteristics of Patients for Overall Survival Analysis

Characteristic^a^	Patients, No (%)					
	Before PSM (n = 1569)			After PSM (n = 990)		
	DR (n = 1057)	SR (n = 512)	*P* value^b^	DR (n = 495)	SR (n = 495)	*P* value^b^
Demographic						
Age, mean (SD), y	57.4 (11.2)	57.1 (10.5)	.66	57.3 (11.2)	57.2 (11.4)	.74
Sex						
Male	719 (68.0)	310 (60.5)	.004	293 (59.2)	296 (59.8)	.90
Female	338 (32.0)	202 (39.5)		202 (40.8)	199 (40.2)	
BMI, mean (SD)	23.1 (3.3)	23.3 (2.8)	.60	23.1 (3.1)	23.2 (2.9)	.67
Tumor sidedness						
Colon	571 (54.0)	392 (76.6)	<.001	373 (75.4)	377 (76.2)	.82
Left	356 (33.7)	171 (33.4)		161 (32.6)	160 (32.4)	
Right	215 (20.3)	221 (43.2)		212 (42.8)	217 (43.8)	
Rectal	486 (46.0)	120 (23.4)		122 (24.6)	118 (23.8)	
Clinical and pathological T stage						
T1-T2	105 (9.9)	49 (9.5)	.82	48 (9.7)	47 (9.6)	>.99
T3-T4	952 (90.1)	463 (90.5)		447 (91.3)	448 (90.4)	
Clinical and pathological N stage						
N0	314 (29.7)	169 (32.9)	.02	145 (29.3)	163 (32.9)	.20
N1	396 (37.4)	210 (41.0)		197 (39.8)	203 (41.0)	
N2	347 (32.8)	133 (26.1)		153 (30.9)	129 (26.1)	
Tumor differentiation						
Poor	296 (28.0)	125 (24.5)	.13	122 (24.6)	125 (25.3)	.83
Moderate or well	761 (72.0)	387 (75.5)		373 (75.4)	370 (74.7)	
Metastatic sites						
Maximum size, cm						
≤5	892 (84.3)	433 (84.6)	.94	443 (89.4)	433 (87.4)	.32
>5	165 (15.7)	79 (15.4)		52 (10.6)	62 (12.6)	
No.						
≤10	933 (88.3)	460 (89.9)	.35	464 (93.8)	446 (90.2)	.05
>10	124 (11.7)	52 (10.1)		31 (6.2)	49 (9.8)	
Distributions						
Unilobar	614 (58.1)	319 (62.2)	.11	294 (59.4)	307 (62.1)	.40
Bilobar	443 (41.9)	193 (37.8)		201 (40.6)	188 (37.9)	
Oligometastatic diseases						
Yes	874 (82.7)	423 (82.6)	>.99	407 (82.3)	410 (82.9)	.80
No	183 (17.3)	89 (17.4)		88 (17.7)	85 (17.1)	
Anticipated hepatectomy						
Minor	784 (74.2)	413 (80.7)	.004	389 (78.6)	396 (80.1)	.64
Major^c^	273 (25.8)	99 (19.3)		106 (21.4)	99 (19.9)	
*KRAS* or *BRAF* status						
*KRAS* wild-type	517 (48.9)	240 (46.9)	.51	241 (48.7)	239 (48.3)	.40
*KRAS* sequence variation	388 (36.7)	204 (39.8)		207 (41.8)	204 (41.2)	
*BRAF* sequence variation	45 (4.3)	16 (3.1)		22 (4.4)	16 (3.2)	
NA	107 (10.1)	52 (10.2)		25 (5.1)	36 (7.3)	
Chemotherapy						
Preoperative chemotherapy	341 (32.3)	241 (47.1)	<.001	202 (40.8)	213 (43.0)	.52
Interval chemotherapy	790 (74.7)	NA		407 (82.3)	NA	
Postoperative chemotherapy	668 (63.2)	333 (65.1)	.50	330 (66.6)	324 (65.4)	.74

Abbreviations: BMI, body mass index (calculated as weight in kilograms divided by height in meters squared); DR, delayed resection; NA, not available; PSM, propensity score matching; SR, simultaneous resection.

^a^ Continuous variables are presented as mean (SD) and categorical variables as No. (%).

^b^ *P* values were calculated using the χ^2^ test for categorical variables and Mann-Whitney test for continuous variables.

^c^ Major hepatectomy is defined as resection of 3 or more liver segments.

In addition, 223 of 1569 patients (14.2%) were excluded from the entire cohort because of death irrelevant to CRLM. A total of 1346 eligible patients were identified for the CSS analysis (eTable 1 in the Supplement).

### Comparison of Perioperative Complications

eTable 2 in the Supplement shows the primary outcome. The percentages of perioperative complications occurring within 60 days of surgery were not statistically different among those who received simultaneous resection or delayed resection for CRC after corresponding unbiased estimates (34.1% vs 30.0%; *P* = .89).

The proportions of digestive complications were 9.3% and hepatic complications were 11.2% in the delayed resection group, whereas the percentages were 11.7% and 12.3%, respectively, in the simultaneous resection group (eTable 3 in the Supplement). We did not observe any intraoperative deaths, but a total of 5 patients in both groups died in the first 3 months of the postoperative period.

According to the Clavien-Dindo classification, the proportions of complications were similar in the 2 groups. The percentages of grades III (requiring surgical, endoscopic, or radiological intervention) and IV (life-threatening [including central nervous system complications] requiring intensive care unit management) complications were 48.8% in the delayed resection group vs 49.0% in the simultaneous resection group. These differences did not reach significance levels (eTable 3 in the Supplement).

### Outcomes of Simultaneous vs Delayed Resection 

**Figure 1.**
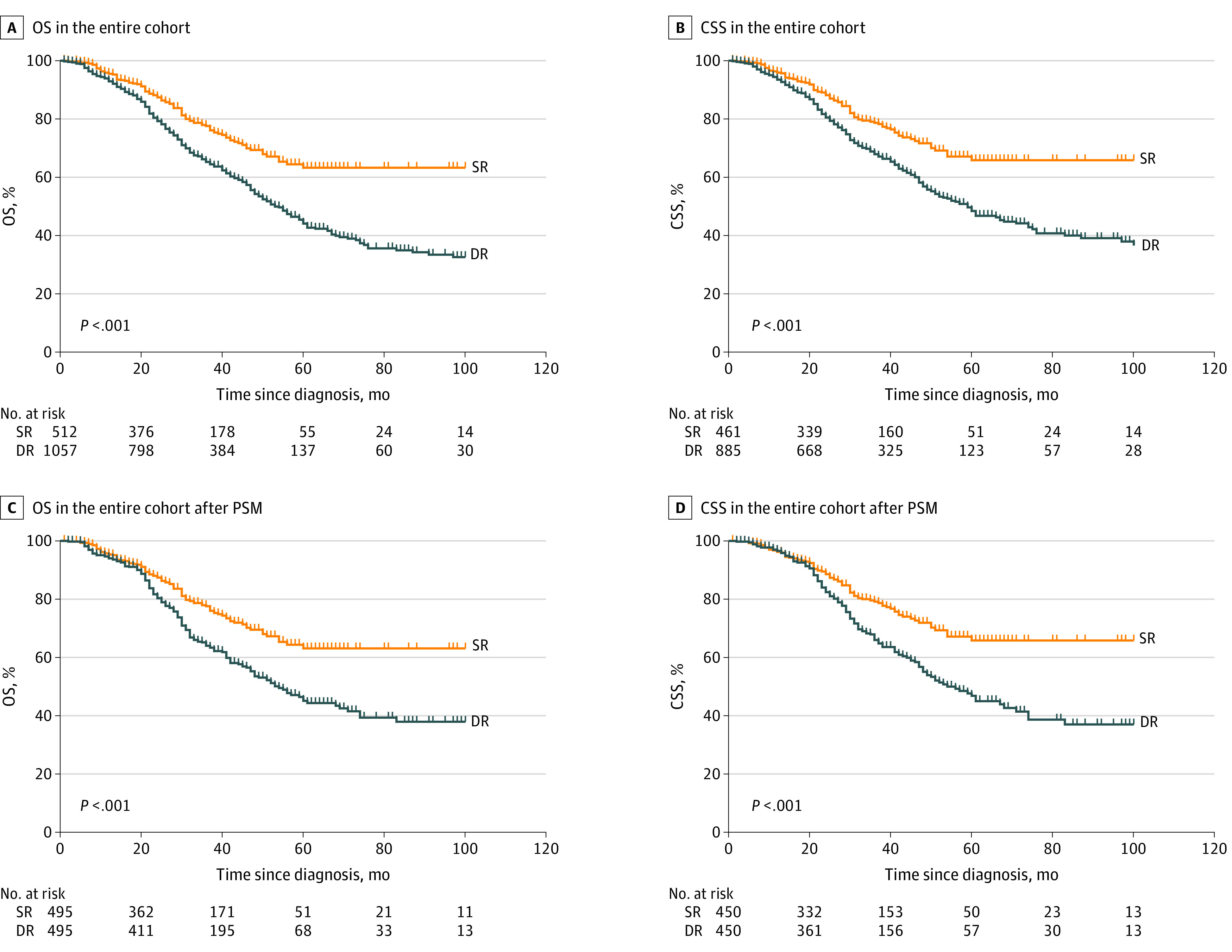
Kaplan-Meier Curves for Overall Survival (OS) and Cancer-Specific Survival (CSS) Stratified by Resection Strategies in the Entire Cohorts DR indicates delayed resection; PSM, propensity score matching; SR, simultaneous resection.

The median (range) follow-up period was 72.3 (6.2-179.3) months in the delayed resection group and 74.5 (17.9-188.2) months in the simultaneous resection group. The OS rates were 67.5% at 3 years, 46.4% at 5 years, and 33.5% at 8 years for the delayed resection group, whereas the OS rates were 78.7% at 3 years, 64.4% at 5 years, and 63.3% at 8 years for the simultaneous resection group (HR, 1.50; 95% CI, 1.21-1.88; *P* < .001) (Figure 1A; Table 2). The CSS rates were 69.8% at 3 years, 50.9% at 5 years, and 39.1% at 8 years for the delayed resection group and were 79.5% at 3 years, 67.2% at 5 years, and 65.8% at 8 years for the simultaneous resection group (HR, 1.45; 95% CI, 1.14-1.86; *P* = .003) (Figure 1B; Table 2).

**Table 2.** Univariate and Multivariate Analyses of Factors Associated With Overall Survival and Cancer-Specific Survival Before and After PSM

Characteristic	Overall survival								Cancer-specific survival							
	Before PSM				After PSM				Before PSM				After PSM			
	Univariate		Multivariate		Univariate		Multivariate		Univariate		Multivariate		Univariate		Multivariate	
	HR (95% CI)	*P* value	HR (95% CI)	*P* value	HR (95% CI)	*P* value	HR (95% CI)	*P* value	HR (95% CI)	*P* value	HR (95% CI)	*P* value	HR (95% CI)	*P* value	HR (95% CI)	*P* value
Treatment																
SR	1 [Reference]	<.001	1 [Reference]	<.001	1 [Reference]	<.001	1 [Reference]	.003	1 [Reference]	<.001	1 [Reference]	.003	1 [Reference]	<.001	1 [Reference]	.004
DR	1.75 (1.42-2.15)		1.50 (1.21-1.88),		1.70 (1.35-2.14)		1.42 (1.10-1.85)		1.73 (1.37-2.17)		1.45 (1.14-1.86)		1.77 (1.37-2.28)		1.50 (1.14-1.98)	
Sex																
Male	1 [Reference]	<.001	1 [Reference]	.002	1 [Reference]	<.001	1 [Reference]	.005	1 [Reference]	.003	1 [Reference]	.007	1 [Reference]	.008	1 [Reference]	.01
Female	1.47 (1.19-1.82)		1.31 (1.10-1.56)		1.52 (1.17-1.98)		1.37 (1.10-1.71)		1.43 (1.13-1.85)		1.31 (1.08-1.60)		1.40 (1.09-1.79)		1.35 (1.02-1.73)	
Perioperative chemotherapy																
Yes	1 [Reference]	.41	NA	NA	1 [Reference]	.99	NA	NA	1 [Reference]	.34	NA	NA	1 [Reference]	.66	NA	NA
No	1.01 (0.85-1.24)				0.99 (0.69-1.24)				0.90 (0.74-1.11)				1.06 (0.82-1.36)			
Tumor sidedness																
Left	1 [Reference]	.77	NA	NA	1 [Reference]	.92	NA	NA	1 [Reference]	.84	NA	NA	1 [Reference]	.96	NA	NA
Right	0.93 (0.77-1.11)				1.00 (0.71-1.27)				0.67 (0.43-0.92)				0.99 (0.76-1.29)			
Clinical and pathological T stage																
T1-2	1 [Reference]	.10	NA	NA	1 [Reference]	.12	NA	NA	1 [Reference]	.04	1 [Reference]	.21	1 [Reference]	.17	NA	NA
T3-4	1.09 (0.91-1.36)				1.29 (0.95-1.66)				1.17 (0.86-1.43)		0.98 (0.79-1.18)		1.15 (0.92-1.45)			
Clinical and pathological N stage																
N0	1 [Reference]	<.001	1 [Reference]	.001	1 [Reference]	<.001	1 [Reference]	<.001	1 [Reference]	<.001	1 [Reference]	.001	1 [Reference]	<.001	1 [Reference]	.001
N1	1.19 (0.96-1.48)		1.15 (0.93-1.42)		1.13 (0.85-1.49)		1.02 (0.74-1.31)		1.24 (0.97-1.64)		1.23 (0.92-1.59)		1.19(0.90-1.70)		1.18 (0.88-1.68)	
N2	1.88 (1.52-2.34)		1.64 (1.36-2.07)		1.89 (1.42-2.37)		1.77 (1.38-2.26)		1.89 (1.48-2.32)		1.74 (1.30-2.20)		2.07(1.50-2.85)		1.94 (1.36-2.78)	
Tumor differentiation																
Moderate or well	1 [Reference]	<.001	1 [Reference]	<.001	1 [Reference]	.04	1 [Reference]	.12	1 [Reference]	.001	1 [Reference]	.004	1 [Reference]	.004	1 [Reference]	.009
Poor	1.43 (1.15-1.79)		1.41 (1.13-1.73)		1.31 (1.02-1.68)		1.27 (0.94-1.67)		1.46 (1.16-1.88)		1.44 (1.13-1.84)		1.51(1.14-1.98)		1.37 (1.04-1.74)	
Metastatic sites																
Maximum size, cm																
≤5	1 [Reference]	.37	NA	NA	1 [Reference]	.90	NA	NA	1 [Reference]	.59	NA	NA	1 [Reference]	.43	NA	NA
>5	1.04 (0.86-1.26)				1.02 (0.83-1.29)				0.82 (0.63-1.06)				1.09 (0.87-1.37)			
No.																
≤10	1 [Reference]	.15	NA	NA	1 [Reference]	.18	NA	NA	1 [Reference]	.29	NA	NA	1 [Reference]	.83	NA	NA
>10	1.07 (0.89-1.32)				1.25 (0.92-1.61)				0.94 (0.76-1.14)				1.01 (0.79-1.32)			
Distributions																
Unilobar	1 [Reference]	<.001	1 [Reference]	<.001	1 [Reference]	<.001	1 [Reference]	<.001	1 [Reference]	<.001	1 [Reference]	<.001	1 [Reference]	<.001	1 [Reference]	<.001
Bilobar	1.88 (1.53-2.35)		1.81 (1.47-2.21)		1.99 (1.54-2.48)		1.95 (1.50-2.44)		1.89 (1.51-2.35)		1.81 (1.37-2.26)		1.98 (1.40-2.81)		1.89 (1.32-2.48)	
Anticipated hepatectomy																
Minor	1 [Reference]	.16	NA	NA	1 [Reference]	.56	NA	NA	1 [Reference]	.26	NA	NA	1 [Reference]	.46	NA	NA
Major	1.06 (0.88-1.31)				1.16 (0.88-1.51)				0.97 (0.78-1.18)				1.13 (0.90-1.41)			
*KRAS* or *BRAF* status																
*KRAS* wild-type	1 [Reference]	<.001	1 [Reference]	.001	1 [Reference]	<.001	1 [Reference]	<.001	1 [Reference]	<.001	1 [Reference]	<.001	1 [Reference]	<.001	1 [Reference]	<.001
*KRAS* sequence variation	1.45 (1.17-1.79)		1.43 (1.16-1.76)		1.78 (1.40-2.29)		1.75 (1.36-2.25)		1.54 (1.25-1.96)		1.48 (1.18-1.89)		1.66 (1.37-2.09)		1.65 (1.35-2.08)	
*BRAF* sequence variation	3.58 (2.00-6.41)		3.24 (1.80-5.85)		3.83 (2.16-8.35)		3.25 (1.94-6.88)		4.66 (2.55-8.54)		4.26 (2.36-7.68)		3.75 (1.82-7.74)		2.64 (1.79-7.39)	

Abbreviations: DR, delayed resection; HR, hazard ratio; NA, not available; PSM, propensity score matching; SR, simultaneous resection.

Propensity score matching yielded 495 pairs of patients for the OS analysis and 450 pairs of patients for the CSS analysis (Table 1; eTable 1 in the Supplement). A statistically significant difference in outcomes in the delayed resection group compared with the simultaneous resection group was also observed in OS (the OS rates were 65.2% at 3 years, 47.1% at 5 years, and 38.0% at 8 years for the delayed resection group and 78.0% at 3 years, 65.4% at 5 years, and 63.1% at 8 years for the simultaneous resection group, *P* < .001) and CSS (the CSS rates were 68.3% at 3 years, 48.5% at 5 years, and 37.1% at 8 years for the delayed resection group and 79.2% at 3 years, 67.2% at 5 years, and 65.9% at 8 years for the simultaneous resection group, *P* < .001) analyses (Figure 1C and D). Simultaneous resection strategy was independently associated with better OS (HR, 1.42; 95% CI, 1.10-1.85; *P* = .003) and CSS rates (HR, 1.50; 95% CI, 1.14-1.98; *P* = .004) (Table 2).

**Figure 2.**
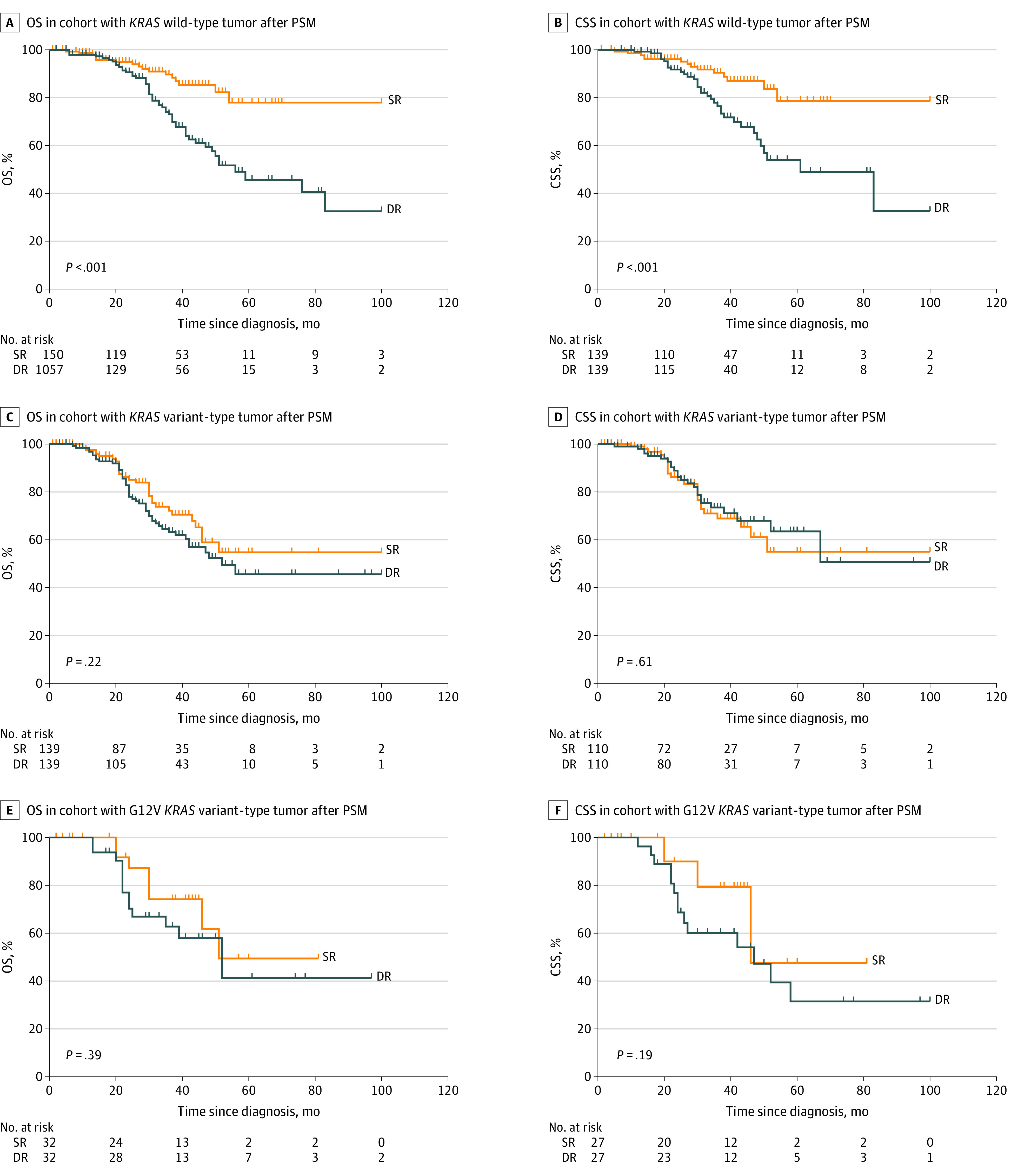
Kaplan-Meier Curves for Overall Survival (OS) and Cancer-Specific Survival (CSS) Stratified by Resection Strategies According to *KRAS* Sequence Variation Status After Propensity Score Matching (PSM) DR indicates delayed resection; SR, simultaneous resection.

In patients with *KRAS* wild-type tumors, propensity score matching yielded 150 pairs of patients for the OS analysis and 139 pairs of patients for the CSS analysis (eTable 4 in the Supplement). For patients with *KRAS* wild-type tumors, simultaneous resection compared with delayed resection was associated with a longer OS rate (5-year OS, 45.7% vs 78.0%; HR, 1.61 [95% CI, 1.45-2.18; *P* < .001]) and CSS rate (5-year CSS, 50.0% vs 78.7%; HR, 1.62 [95 CI, 1.40-1.87; *P* = .003]) (Figure 2A and B).

In patients with overall *KRAS* sequence variation, propensity score matching yielded 139 pairs of patients for the OS analysis and 110 pairs of patients for the CSS analysis (eTable 5 in the Supplement). The outcomes were not statistically different among those who underwent simultaneous liver resection and those who underwent delayed liver resection, as indicated by similar OS rates (HR, 1.23; 95% CI, 0.94-1.51; *P* = .22) and CSS (HR, 0.84; 95% CI, 0.53-1.21; *P* = .73) (Figure 2C and D; Table 3).

**Table 3.** Univariate and Multivariate Analyses of Overall Survival and Cancer-Specific Survival by *KRAS* Sequence Variation Status With PSM

Characteristic	Overall survival								Cancer-specific survival							
	*KRAS* wild-type (n = 300)				*KRAS* sequence variation (n = 278)				*KRAS* wild-type (n = 278)				*KRAS* sequence variation (n = 220)			
	Univariate		Multivariate		Univariate		Multivariate		Univariate		Multivariate		Univariate		Multivariate	
	HR (95% CI)	*P* value	HR (95% CI)	*P* value	HR (95% CI)	*P* value	HR (95% CI)	*P* value	HR (95% CI)	*P* value	HR (95% CI)	*P* value	HR (95% CI)	*P* value	HR (95% CI)	*P* value
Treatment																
SR	1 [Reference]	<.001	1 [Reference]	<.001	1 [Reference]	.22	NA	NA	1 [Reference]	.002	1 [Reference]	.003	1 [Reference]	.73	NA	NA
DR	1.72 (1.57-2.30)		1.61 (1.45-2.18)		1.23 (0.94-1.51)				1.65 (1.43-1.91)		1.62 (1.40-1.87)		0.84 (0.53-1.21)			
Sex																
Male	1 [Reference]	<.001	1 [Reference]	.005	1 [Reference]	.35	NA	NA	1 [Reference]	.001	1 [Reference]	.003	1 [Reference]	.75	NA	NA
Female	1.68 (1.54-2.24)		1.46 (1.30-2.03)		1.16 (0.85-1.40)				1.67 (1.45-1.94)		1.61 (1.39-1.87)		0.82 (0.51-1.19)			
Perioperative chemotherapy																
Yes	1 [Reference]	.57	NA	NA	1 [Reference]	.59	NA	NA	1 [Reference]	.50	NA	NA	1 [Reference]	.54	NA	NA
No	0.76 (0.53-1.14)				1.03 (0.72-1.28)				1.02 (0.79-1.30)				1.04 (0.74-1.38)			
Tumor sidedness																
Left	1 [Reference]	.17	NA	NA	1 [Reference]	.92	NA	NA	1 [Reference]	.56	NA	NA	1 [Reference]	.99	NA	NA
Right	1.20 (1.00-1.57)				0.58 (0.32-0.84)				0.98 (0.76-1.26)				0.61 (0.36-0.97)			
Clinical and pathological T stage																
T1-2	1 [Reference]	.86	NA	NA	1 [Reference]	.21	NA	NA	1 [Reference]	.90	NA	NA	1 [Reference]	.21	NA	NA
T3-4	0.53 (0.39-1.01)				1.24 (0.95-1.53)				0.55 (0.33-0.83)				1.31 (1.05-1.66)			
Clinical and pathological N stage														.		
N0	1 [Reference]	.04	1 [Reference]	.18	1 [Reference]	.04	1 [Reference]	.12	1 [Reference]	.44	NA	NA	1 [Reference]	.03	1 [Reference]	.18
N1	0.85 (0.64-1.23		0.77 (0.54-1.15)		0.90 (0.60-1.11)		1.36 (1.06-1.67)		0.89 (0.64-1.18)				1.08 (0.81-1.41)		0.98 (0.76-1.37)	
N2	1.35 (1.17-1.74)		1.27 (1.09-1.67)		1.45 (1.16-1.75)				1.17 (0.94-1.42)				1.50 (1.25-1.82)		1.40 (1.14-1.73)	
Tumor differentiation																
Moderate or well	1 [Reference]	<.001	1 [Reference]	.001	1 [Reference]	.002	1 [Reference]	.008	1 [Reference]	.10	1 [Reference]	.02	1 [Reference]	.001	1 [Reference]	.01
Poor	1.69 (1.55-2.26)		1.49 (1.34-2.07)		1.64 (1.33-1.92)		1.55 (1.24-1.80)		1.39 (1.16-1.74)		1.32 (1.11-1.69)		1.58 (1.31-2.02)		1.45 (1.20-1.81)	
Metastatic sites																
Maximum size, cm																
≤5	1 [Reference]	.54	NA	NA	1 [Reference]	.44	NA	NA	1 [Reference]	.68	NA	NA	1 [Reference]	.84	NA	NA
>5	0.73 (0.51-1.11)				1.09 (0.78-1.34)				0.85 (0.59-1.13)				0.76 (0.46-1.08)			
No.																
≤10	1 [Reference]	.02	1 [Reference]	.04	1 [Reference]	.13	NA	NA	1 [Reference]	.58	NA	NA	1 [Reference]	.57	NA	NA
>10	1.25 (1.07-1.65)		1.23 (1.04-1.63)		1.37 (1.08-1.69)				0.95 (0.73-1.24)				0.92 (0.71-1.35)			
Distributions																
Unilobar	1 [Reference]	<.001	1 [Reference]	.004	1 [Reference]	.10	NA	NA	1 [Reference]	.06	NA	NA	1 [Reference]	.06	NA	NA
Bilobar	1.71 (1.55-2.29)		1.47 (1.31-2.04)		1.34 (1.04-1.64)				1.23 (1.03-1.51)				1.39 (1.13-1.73)			
Anticipated hepatectomy																
Minor	1 [Reference]	.10	NA	NA	1 [Reference]	.23	NA	NA	1 [Reference]	.38	NA	NA	1 [Reference]	.24	NA	NA
Major	1.13 (0.94-1.50)				1.45 (1.16-1.77)				0.73 (0.54-1.06)				1.33 (1.04-1.56)			

Abbreviations: DR, delayed resection; HR, hazard ratio; NA, not available; PSM, propensity score matching; SR, simultaneous resection.

The extent to which specific sequence variations were associated with the biological behavior of *KRAS* appeared to be different as well. We sought to stratify the advantages of resection strategies according to the G12V *KRAS* sequence variation. Among patients with *KRAS* sequence variations, the G12V variant was observed in 87 patients (22.4%) in the delayed resection group and 54 patients (26.5%) in the simultaneous resection group in the OS cohort. In the CSS cohort, the G12V variant was observed in 75 patients (22.4%) in the delayed resection group and 50 patients (30.3%) in the simultaneous resection group (eTable 6 in the Supplement).

Propensity score matching yielded 32 pairs of patients for the OS analysis and 27 pairs of patients for the CSS analysis (eTable 7 in the Supplement). We found that patients with the G12V *KRAS* allele exhibited similar OS rates (HR, 1.21; 95% CI, 0.96-1.52; *P* = .38) and CSS (HR, 1.23; 95% CI, 1.02-1.57; *P* = .20) regardless of the treatment received (Figure 2E and F; eTable 8 in the Supplement).

## Discussion

In this multiinstitutional analysis of patients with resected CRLM, the percentage of perioperative complications did not differ whether resection of the CRC and SLM was simultaneous or delayed. Simultaneous resection was associated with improved survival outcomes both in the overall cohort and in the subset of patients with *KRAS* wild-type tumors. However, patients with tumors with *KRAS* sequence variation exhibited similar outcomes, regardless of the resection strategy. These findings suggest that patients with CRC and initially resectable SLM could undergo simultaneous resection. To our knowledge, this study was the first to classify the underlying benefits of simultaneous vs delayed resection of liver metastases by incorporating *KRAS* sequence variation data. The findings may inform arguments for change by clinical teams who, in the absence of evidence-based data, rely on the precautionary principle of choosing delayed resection of liver metastases from CRC.^20,21,22^

The timing of liver resection for liver metastases in CRC has been a long-standing topic of debate given that several studies have reported conflicting results associated with either approach.^23^ The debate continues primarily because of the lack of randomization, and studies depend on the condition of tumors, general condition of patients, desire of patients, and surgical team availability; therefore, comparisons between studies vary. To our knowledge, the study population of 1569 individual patients is the largest to date. We performed propensity score matching of the clinical characteristics of the 2 groups and placed patients in both groups in similar favorable conditions given that their clinical features were well balanced. Delayed resection displayed inferior 10-year OS and CSS rates compared with simultaneous resection. This result may be attributed to the progression of liver metastases after colorectal surgery and suggests a liver-first approach. However, combining the delayed resection strategy with the liver-first approach could circumvent the risk of liver disease progression, but it may not be able to limit extrahepatic progression.^1^ Three systematic reviews comparing the liver-first, colorectal-first, and simultaneous resection approaches were unable to identify a clear survival benefit in terms of 5-year OS for any of the 3 strategies.^1,24,25^ As such, randomized clinical trials should further explore the optimal role and timing of liver resection in patients with SLM. Furthermore, it raises the question of whether the time interval between the 2 stages allowed time for disease progression. The precise definition of the optimal time interval between the 2 operations is lacking. We chose a time interval of 15 to 60 days on the basis of previous experience and to avoid complications at the second operation. Currently, we suggest that patients undergo liver resection for liver metastases as soon as they have recovered from the first operation.

Colorectal cancer exhibits high heterogeneity,^26^ with molecularly defined subgroups who differ in their outcomes.^27,28,29^ In this study, we discovered that the advantages of the simultaneous resection strategy was restricted to patients with *KRAS* wild-type tumors. Moreover, the G12V *KRAS* sequence variations are the most forms of the *KRAS *variant subtypes that reflect the aggressiveness of *KRAS*-derived malignant cancer.^30^ Similarly, patients with the G12V variant did not exhibit superior outcomes with simultaneous or delayed hepatectomy. This finding is important given that *KRAS* sequence variations have been shown to be clinically relevant biomarkers associated with the therapeutic benefits of certain cancer treatments and to have implications for patient outcome.^12,13,31^

We hypothesized that tumor biology may have confounded previous analyses and that biological factors may directly affect tumor growth patterns and consequently the oncologic benefits of resection strategies. Simultaneous resection was no longer a statistically significant factor in the multivariate analysis after molecular marker was incorporated into the analysis. This result suggests that clinical outcome was more closely associated with tumor biology than resection strategies. The presence of *KRAS* sequence variation, one of the most common surrogates of tumor biological factors, appeared to trigger a distinct pattern of intrahepatic tumor growth and dissemination,^32^ which may affect the beneficial outcome of simultaneous resection in this patient subgroup. Thus, it may be reasonable to hypothesize that liver metastatic burden will be successfully managed by simultaneous resection and contribute to achieved survival in contrast with patients with *KRAS* wild-type tumors.

Findings of this study suggest that simultaneous resection may be recommended for patients with *KRAS* wild-type tumors. The data support the ideal that molecular status should be known and considered in the decision-making process before surgery. Integrating molecular features in the choice of treatment provides a theoretical basis for more accurate individualized treatments.

### Limitations

This study has several limitations. First, we used a retrospective approach; thus, the study is inherently flawed by selection and indication biases. Second, although we performed propensity score matching and multivariate analysis to enhance intergroup comparison, unidentified biases may have acted in favor of the simultaneous resection group. Third, extended *RAS* sequence variation beyond *KRAS* sequence variations and other factors, including *TP53*, *SMAD4*, and *BRAF* alterations, were not included because of limited availability of data. The advantages of simultaneous resection in CRLM with specific driver sequence variations could be more profound.

## Conclusions

To our knowledge, this comparative effectiveness research study is the first to find that the survival benefits of simultaneous resection were restricted to patients with *KRAS* wild-type tumors. It is acceptable for patients presenting with CRC and resectable SLM to undergo simultaneous resection of the primary tumor and the liver metastases. Integrating molecular features in the choice of treatment before surgery provides a theoretical basis for more accurate individualized treatments.

## Notes

Supplement.
eFigure.
**eTable 1.** Patient Characteristics of Patients for Cancer-Specific Survival Analysis **eTable 2.** Numbers and Percentages of Patients Who Had At Least One Major Complications After Corresponding Unbiased Estimates **eTable 3.** Type and Number of Major Complications After Corresponding Unbiased Estimates **eTable 4.** Patient Characteristics by Treatment in *KRAS*-WT Patients **eTable 5.** Patient Characteristics by Treatment in *KRAS*-MT Patients **eTable 6.** The Distributions of *KRAS* Variant Subtypes **eTable 7.** Patient Characteristics by Treatment in G12V *KRAS*-MT Patients **eTable 8.** Univariate and Multivariate Analyses in Overall Survival and Cancer-Specific Survival in G12V *KRAS-*MT Patients With PSM
