# Supplementary material for: Association of Simultaneous vs Delayed Resection of Liver Metastasis With Complications and Survival Among Adults With Colorectal Cancer
Source: JAMA Netw Open. 2022 Sep 19;5(9):e2231956. doi: 10.1001/jamanetworkopen.2022.31956 (PMC9486453; doi:10.1001/jamanetworkopen.2022.31956)
Supplement: Supplement. — eFigure. eTable 1. Patient Characteristics of Patients for Cancer-Specific Survival Analysis eTable 2. Numbers and Percentages of Patients Who Had At Least One Major Complications After Corresponding Unbiased Estimates eTable 3. Type and Number of Major Complications After Corresponding Unbiased Estimates eTable 4. Patient Characteristics by Treatment in KRAS-WT Patients eTable 5. Patient Characteristics by Treatment in KRAS-MT Patients eTable 6. The Distributions of KRAS Variant Subtypes eTable 7. Patient Characteristics by Treatment in G12V KRAS-MT Patients eTable 8. Univariate and Multivariate Analyses in Overall Survival and Cancer-Specific Survival in G12V KRAS-MT Patients With PSM [file jamanetwopen-e2231956-s001.pdf]

Supplemental Online Content

Wu Y, Mao A, Wang H, et al. Association of simultaneous vs delayed resection of liver metastasis with complications and survival among adults with colorectal cancer. *JAMA Netw Open*. 2022;5(9):e2231956. doi:10.1001/jamanetworkopen.2022.31956

eFigure.

eTable 1. Patient Characteristics of Patients for Cancer-Specific Survival Analysis

eTable 2. Numbers and Percentages of Patients Who Had At Least One Major Complications After Corresponding Unbiased Estimates

eTable 3. Type and Number of Major Complications After Corresponding Unbiased Estimates

eTable 4. Patient Characteristics by Treatment in *KRAS*-WT Patients

eTable 5. Patient Characteristics by Treatment in *KRAS*-MT Patients

eTable 6. The Distributions of *KRAS* Variant Subtypes

eTable 7. Patient Characteristics by Treatment in G12V *KRAS*-MT Patients

eTable 8. Univariate and Multivariate Analyses in Overall Survival and Cancer-Specific Survival in G12V *KRAS*-MT Patients With PSM

This supplemental material has been provided by the authors to give readers additional information about their work.

© 2022 Wu Y et al. *JAMA Network Open.*

eFigure.

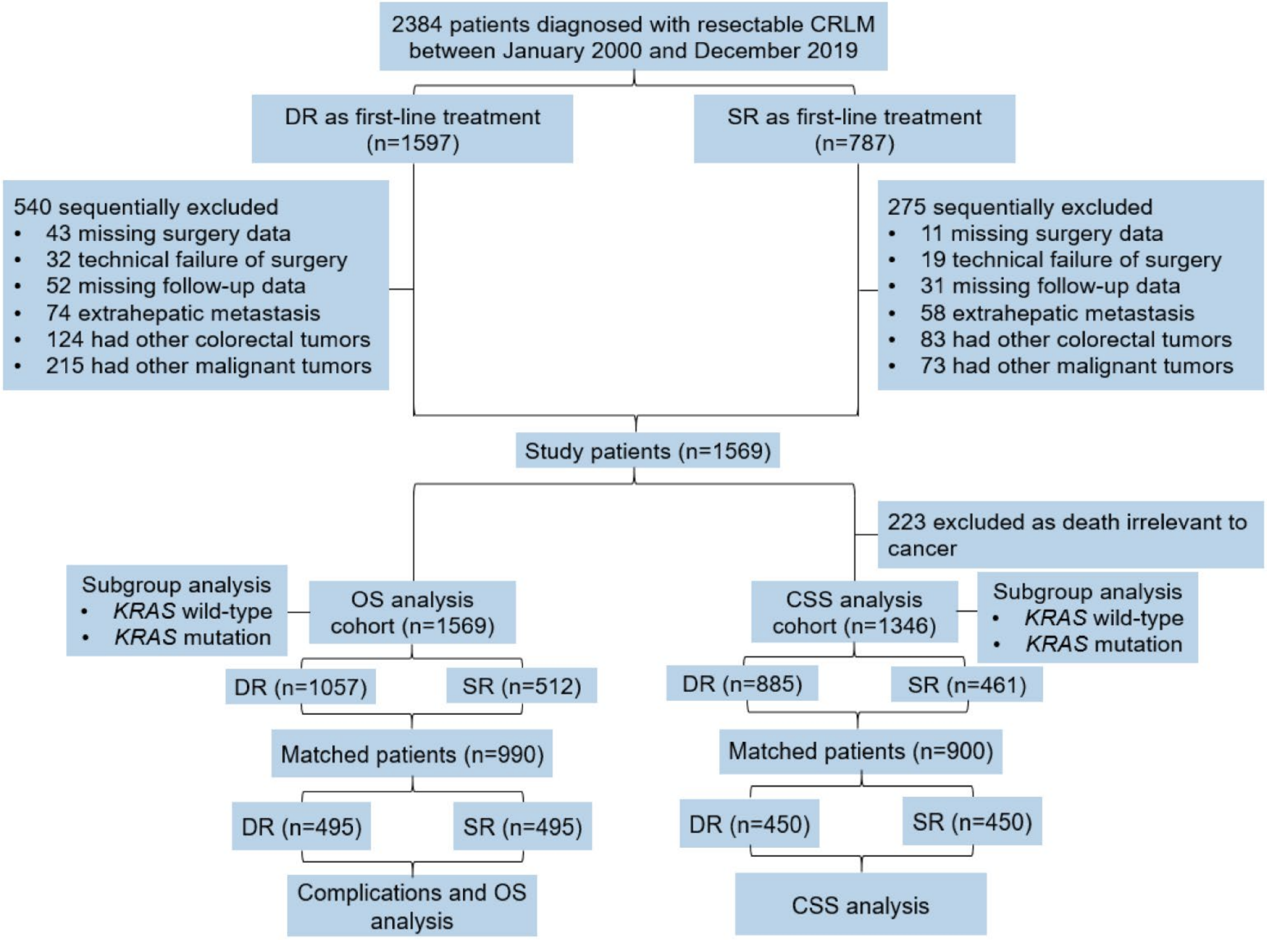

© 2022 Wu Y et al. *JAMA Network Open.*

eTable 1. Patient Characteristics of Patients for Cancer-Specific Survival Analysis

Characteristic [a]	Patients, No (%)					
	Before PSM (n=1346)			After PSM (n=900)		
	DR (n=885)	SR (n=461)	*P* value [b]	DR (n=450)	SR (n=450)	*P* value [b]
Sex						
Male	625 (70.6)	283 (61.4)	＜.001	272 (60.4)	283 (62.9)	.45
Female	260 (29.4)	178 (38.6)		178 (39.6)	167 (37.1)	
Age at diagnosis, y						
＜50	197 (22.3)	105 (22.8)	.83	101 (22.4)	103 (22.9)	.87
⩾50	688 (77.7)	356 (77.2)		349 (77.6)	347 (72.1)	
Tumor sidedness						
Left sidedness	688 (77.7)	291 (63.1)	＜.001	307 (68.3)	291 (64.7)	.29
Right sidedness	197 (22.3)	170 (36.9)		143 (31.7)	159 (35.3)	
c/p T stage						
T1-T2	89 (10.0)	45 (9.7)	.92	42 (9.4)	43 (9.5)	＞.99
T3-T4	796 (90.0)	416 (90.3)		408 (90.6)	407 (90.5)	
c/p N stage						
N0	274 (30.9)	150 (32.6)	.08	136 (30.2)	146 (32.5)	.42
N1	323 (36.5)	188 (40.8)		177 (39.4)	184 (41.0)	
N2	288 (32.6)	123 (26.6)		138 (30.4)	120 (26.5)	
Tumor differentiation						
Poor	258 (29.1)	106 (23.1)	.02	113 (25.1)	105 (23.3)	.53
Moderate/well	627 (70.9)	355 (76.9)		337 (74.9)	345 (76.7)	
Metastatic sites						

© 2022 Wu Y et al. *JAMA Network Open.*

Maximum size						
≤5cm	755 (85.3)	387 (83.9)	.52	382 (84.9)	376 (83.8)	.65
＞5cm	130 (14.7)	74 (16.1)		68 (15.1)	74 (16.2)	
Numbers						
≤10	777 (87.8)	417 (90.4)	.14	399 (88.7)	406 (90.2)	.45
＞10	108 (12.1)	44 (9.6)		51 (11.3)	44 (9.8)	
Distributions						
Unilobar	514 (58.1)	288 (62.5)	.12	268 (59.5)	280 (62.2)	.41
Bilobar	371 (41.9)	173 (37.5)		182 (40.5)	170 (37.8)	
Anticipated hepatectomy			.03			.21
Minor hepatectomy	649 (73.3)	363 (78.7)		337 (74.9)	353 (78.4)	
Major hepatectomy [c]	236 (26.7)	98 (21.3)		113 (25.1)	97 (21.6)	
KRAS/BRAF status						
KRAS wild-type	420 (47.5)	230 (49.9)	.74	220 (48.9)	224(49.7)	.91
KRAS mutation	334 (37.7)	164 (35.6)		167 (37.1)	162 (36.1)	
BRAF mutation	42 (4.8)	18 (4.0)		20 (4.4)	17 (3.8)	
NA	89 (10.0)	48 (10.5)		43 (9.6)	47 (10.4)	
Chemotherapy						
Preoperative chemotherapy	287 (32.4)	212 (46.0)	.02	190 (42.2)	201 (44.7)	.50
Interval chemotherapy	676 (76.4)	NA		362 (80.5)	NA	
Postoperative chemotherapy	597 (67.5)	301 (65.3)	.43	303 (67.3)	299 (66.4)	.78

Abbreviations: PSM, propensity score matching; DR, delayed resection; SR, simultaneous resection; y, years; c/p, clinically and pathologically; T, tumor; N, nodes; NA, not available.

a Categorical variables were presented as No. (%).

b *P* values were calculated using the χ^2 test for categorical variables.

c Major hepatectomy is defined as resection of three or more liver segments.

© 2022 Wu Y et al. *JAMA Network Open.*

eTable 2. Numbers and Percentages of Patients Who Had At Least One Major Complications After Corresponding Unbiased Estimates

Characteristic [a]	DR<br>n=495	SR<br>n=495	*P* value [b]
Major complications	148 (30.0)	169 (34.1)	.89
Colon	120 (24.3)	135 (27.2)	
Rectum	28 (5.6)	34 (6.8)	

Abbreviations: DR, delayed resection; SR, simultaneous resection.

a Categorical variable as No. (%).

b *P* values were calculated using the χ2 test for categorical variables.

eTable 3. Type and Number of Major Complications After Corresponding Unbiased Estimates

**Complication types** [a]	**DR (n=495)**	**SR (n=495)**	***P* value** [b]
Digestive complications	46 (9.3)	58 (11.7)	.35
Hemorrhage	5	10	
Peritonitis	14	23	
Intra-abdominal abscess	12	10	
Anastomosis fistula	5	10	
Bowel obstruction	10	5	
Hepatic complications	55 (11.2)	61 (12.3)	.28
Hemorrhage	10	0	
Bile leakage	14	19	
Sub-phrenic abscess	26	42	
Severe liver failure	5	0	
General complications	47 (9.4)	50 (10.1)	.74
Perioperative death	5	5	
Pulmonary infection	5	10	
Wound abscess	32	30	
Severe sepsis or septic shock	5	5	
Acute renal insufficiency	0	0	
Clavien-Dindo Classification [c]	n=167	n=179	.97
Grade I	50 (29.9)	55 (30.9)	
Grade II	30 (18.1)	31 (17.4)	
Grade III	71 (42.5)	78 (43.6)	
IIIa	34 (20.5)	37 (20.8)	
IIIb	37 (22.0)	41 (22.8)	
Grade IV	11 (6.3)	10 (5.4)	
IVa	3 (1.6)	5 (2.7)	
IVb	8 (4.7)	5 (2.7)	
Grade V	5 (3.1)	5 (2.7)	

Abbreviation: DR, delayed resection; SR, simultaneous resection.

a Categorical variables were presented as No. (%).

b *P* values were calculated using the χ^2 test for categorical variables.

c In DR group, we only considered the highest type of the 2 procedures.

© 2022 Wu Y et al. *JAMA Network Open.*

eTable 4. Patient Characteristics by Treatment in *KRAS*-WT Patients

Characteristic [a]	Patients, No (%)							
	OS cohort				CSS cohort			
	Before PSM (n=757)		After PSM (300)		Before PSM (n=650)		After PSM (n=278)	
	DR (n=517)	SR (n=240)	DR (n=150)	SR (n=150)	DR (n=420)	SR (n=230)	DR (n=139)	SR (n=139)
Sex	.06		.90		.008		.52	
Male	380 (73.6)	160 (66.7)	102 (67.7)	100 (66.7)	319 (75.9)	152 (66.2)	98 (70.5)	92 (66.2)
Female	137 (26.4)	80 (33.3)	48 (32.3)	50 (33.3)	101 (24.1)	78 (33.8)	41 (29.5)	47 (33.8)
Age at diagnosis, y	.17		> .99		.71		.51	
< 50	135 (26.1)	51 (21.4)	40 (26.7)	40 (26.7)	110 (26.3)	64 (27.7)	44 (31.7)	38 (27.3)
≥50	382 (73.9)	189 (78.6)	110 (73.3)	110 (73.3)	310 (73.7)	166 (72.3)	95 (68.3)	101 (72.7)
Tumor sidedness	< .001		.53		.05		.16	
Left sidedness	458 (88.6)	182 (76.0)	129 (86.0)	124 (82.7)	376 (89.5)	193 (83.9)	125 (89.9)	116 (83.5)
Right sidedness	59 (11.4)	58 (24.0)	21 (14.0)	26 (17.3)	44 (10.5)	37 (16.1)	14 (10.1)	23 (16.5)
Perioperative chemotherapy	.43		.82		.01		.55	
Yes	220 (42.5)	110 (46.0)	78 (52.0)	81 (54.0)	180 (42.9)	122 (53.0)	59 (42.4)	65 (46.8)
No	297 (57.5)	130 (54.0)	72 (48.0)	69 (46.0)	240 (57.1)	108 (47.0)	80 (57.6)	74 (53.2)
c/p T stage	.71		.85		0.46		.86	
T1-T2	60 (11.5)	25 (10.3)	17 (11.6)	15 (10.3)	55 (13.0)	25 (10.9)	20 (14.4)	18 (12.9)
T3-T4	457 (88.5)	215 (89.7)	133 (88.4)	135 (89.7)	365 (87.0)	205 (89.1)	119 (85.5)	121 (87.0)
c/p N stage	.30		.18		.30		.62	
N0	144 (27.8)	66 (27.4)	41 (27.5)	40 (26.9)	123 (29.3)	62 (27.0)	44 (31.6)	38 (27.0)
N1	203 (39.2)	107 (44.5)	53 (35.6)	67 (44.8)	164 (39.1)	104 (45.3)	62 (44.4)	63 (45.3)
N2	170 (33.0)	67 (27.9)	55 (36.9)	42 (28.3)	133 (31.6)	64 (27.7)	33 (24.1)	39 (27.7)

© 2022 Wu Y et al. *JAMA Network Open.*

Tumor differentiation	.15		＞.99		.30		.78	
Poor	140 (27.1)	53 (21.9)	46 (30.8)	45 (29.9)	118 (28.2)	49 (21.3)	37 (26.9)	34 (24.3)
Moderate/well	377 (72.9)	187 (78.1)	104 (69.2)	105 (70.1)	302 (71.8)	181 (78.7)	102 (73.4)	105 (75.5)
Metastatic sites								
Maximum size	.12		＞.99		.06		.77	
≤5cm	419 (81.1)	206 (85.8)	115 (76.4)	116 (77.6)	355 (81.7)	186 (80.9)	111 (80.2)	108 (77.9)
＞5cm	98 (18.9)	34 (14.2)	35 (23.6)	34 (22.4)	65 (18.3)	44 (19.1)	28 (19.8)	31 (22.1)
Numbers	.26		＞.99		.27		.71	
≤10	456 (88.2)	219 (91.3)	134 (89.5)	133 (89.0)	356 (84.8)	205 (89.1)	121 (87.0)	124 (89.0)
＞10	61 (11.8)	21 (18.7)	16 (10.5)	17 (11.0)	64 (15.2)	25 (10.9)	18 (13.0)	15 (10.8)
Distributions	.43		.91		.15		.23	
Unilobar	259 (50.0)	128 (53.3)	88 (58.6)	90 (60.3)	219 (52.1)	140 (61.0)	74 (53.2)	85 (61.0)
Bilobar	258 (50.0)	112 (46.7)	62 (41.3)	60 (39.7)	201 (47.9)	90 (39.0)	65 (46.8)	54 (39.0)
Anticipated hepatectomy	.18		.69		.04		.78	
Minor hepatectomy	381 (73.6)	188 (78.2)	112 (74.5)	116 (77.3)	311(74.1)	180(78.2)	105 (75.3)	108 (77.4)
Major hepatectomy [b]	136 (26.4)	52 (21.8)	38 (25.5)	34 (22.7)	109(25.9)	50(21.8)	34 (24.7)	31 (22.6)

Abbreviations: WT, wild-type; PSM, propensity score matching; DR, delayed resection; SR, simultaneous resection; y, years; c/p, clinically and pathologically; T, tumor; N, nodes.

a Categorical variables were presented as No. (%).

b Major hepatectomy is defined as resection of three or more liver segments.

eTable 5. Patient Characteristics by Treatment in *KRAS*-MT Patients

Characteristic [a]	Patients, No (%)							
	OS cohort				CSS cohort			
	Before PSM (n=592)		After PSM (n=278)		Before PSM (n=498)		After PSM (n=220)	
	DR (n=388)	SR (n=204)	DR (n=139)	SR (n=139)	DR (n=334)	SR (n=164)	DR (n=110)	SR (n=110)
Sex	> .99		.81		.37		> .99	
Male	236 (60.8)	124 (60.9)	82 (58.6)	85 (60.9)	224 (67.2)	103 (62.7)	70 (63.6)	69 (62.7)
Female	152 (39.2)	80 (39.1)	57 (41.4)	54 (39.1)	110 (32.8)	61 (37.3)	40 (36.4)	41 (37.3)
Age at diagnosis, y	.006		.79		.21		> .99	
< 50	80 (20.7)	63 (30.8)	40 (28.5)	43 (30.8)	54 (16.3)	34 (20.7)	23 (20.9)	24 (21.8)
≥50	308 (79.3)	141 (69.2)	99 (71.5)	96 (69.2)	280 (83.7)	130 (79.3)	87 (79.1)	86 (78.2)
Tumor sidedness	< .001		.62		< .001		.78	
Left sidedness	294 (75.9)	109 (53.4)	86 (62.1)	81 (58.3)	257 (76.8)	98 (59.8)	47 (42.4)	44 (40.0)
Right sidedness	94 (24.1)	95 (46.6)	53 (38.9)	58 (41.7)	77 (23.2)	66 (40.2)	63 (57.6)	66 (60.0)
Perioperative chemotherapy	.002		> .99		.02		> .99	
Yes	121 (31.2)	90 (44.4)	62 (44.4)	62 (44.4)	107 (32.1)	70 (42.7)	47 (42.7)	47 (42.7)
No	267 (68.8)	114 (55.6)	77 (55.6)	77 (55.6)	227 (67.9)	94 (57.3)	63 (57.3)	63 (57.3)
c/p T stage	> .99		> .99		.46		.78	
T1-T2	28 (7.2)	15 (7.3)	9 (6.5)	10 (7.1)	26 (7.7)	9 (5.7)	8 (7.7)	6 (5.7)
T3-T4	360 (92.8)	189 (92.7)	130 (93.5)	129 (92.9)	308 (92.3)	155 (94.3)	102 (92.3)	104 (94.5)
c/p N stage	.70		.96		.23		.88	
N0	101 (26.1)	59 (29.1)	38 (27.4)	40 (29.1)	88 (26.4)	33 (28.0)	34 (30.8)	31 (28.0)
N1	164 (42.3)	80 (39.4)	56 (40.2)	55 (39.4)	134 (40.1)	77 (47.0)	41 (37.5)	41 (37.4)

© 2022 Wu Y et al. *JAMA Network Open.*

N2	122 (31.5)	65 (31.5)	45 (32.4)	44 (31.5)	112 (33.5)	54 (33.0)	35 (31.8)	38 (34.5)
Tumor differentiation	.03		.50		.59		.87	
Poor	136 (35.0)	53 (26.0)	42 (30.0)	36 (26.0)	90 (27.0)	40 (24.5)	29 (26.2)	27 (24.5)
Moderate/well	252 (65.0)	151 (74.0)	97 (69.8)	103 (74.0)	244 (73.0)	124 (75.5)	81 (73.8)	83 (75.5)
Metastatic sites								
Maximum size	.82		.75		.72		.86	
≤5cm	323 (83.2)	168 (82.2)	117 (84.2)	114 (82.2)	269 (80.4)	135 (82.2)	88 (80.2)	90 (82.2)
＞5cm	65 (16.8)	36 (17.8)	22 (15.8)	25 (17.8)	65 (19.6)	29 (17.8)	22 (20.0)	20 (18.2)
Numbers	.71		.88		.67		.85	
≤10	331 (85.4)	177 (86.8)	113 (81.5)	115 (82.9)	290 (86.8)	145 (88.4)	94 (85.1)	92 (83.2)
＞10	57 (14.6)	27 (13.2)	26 (19.5)	24 (17.1)	44 (13.2)	19 (11.6)	16 (14.5)	18 (16.8)
Distributions	.43		.90		.33		.78	
Unilobar	224 (57.8)	125 (61.3)	82 (58.9)	80 (57.2)	215 (64.3)	98 (60.0)	70 (63.9)	67 (60.5)
Bilobar	164 (42.2)	79 (38.7)	57 (41.1)	59 (42.8)	119 (35.6)	66 (40.0)	40 (36.1)	43 (39.5)
Anticipated hepatectomy	.11		.68		.19		.75	
Minor hepatectomy	281 (73.2)	160 (78.4)	103 (74.3)	107 (77.2)	241 (72.2)	128 (77.9)	82 (74.7)	85 (77.1)
Major hepatectomy [b]	107 (26.8)	44 (21.6)	36 (25.7)	32 (22.8)	93 (27.8)	36 (22.1)	28 (25.3)	25 (22.9)

Abbreviations: MT, mutant-type; OS, overall survival; CSS, cancer-specific survival; PSM, propensity score matching; DR, delayed resection; SR, simultaneous resection; y, years; c/p, clinically and pathologically; T, tumor; N, node.

a Categorical variables were presented as No. (%).

b Major hepatectomy is defined as resection of three or more liver segments.

© 2022 Wu Y et al. *JAMA Network Open.*

eTable 6. The Distributions of *KRAS* Variant Subtypes

Subtypes	Patients, No (%)			
	OS cohort		CSS cohort	
	DR (n=388)	SR (n=204)	DR (n=334)	SR (n=164)
G12D	127 (32.8)	75 (36.6)	109 (32.5)	56 (33.9)
G12V	87 (22.4)	54 (26.5)	75 (22.4)	50 (30.3)
G12C	19 (4.8)	3 (1.5)	17 (5.0)	3 (1.8)
G12A	10 (2.5)	4 (2.2)	8 (2.4)	5 (2.8)
G12X	34 (8.7)	12 (6.0)	33 (9.9)	12 (7.3)
G12S	11 (2.8)	11 (5.2)	11 (3.2)	8 (4.6)
G13D	52 (13.3)	38 (18.7)	48 (14.4)	24 (14.7)

Abbreviations: OS, overall survival; CSS, cancer-specific survival; DR, delayed resection; SR, simultaneous resection.

© 2022 Wu Y et al. *JAMA Network Open.*

eTable 7. Patient Characteristics by Treatment in G12V *KRAS*-MT Patients

Characteristic [a]	Patients, No (%)							
	OS cohort				CSS cohort			
	Before PSM (n=141)		After PSM (n=64)		Before PSM (n=125)		After PSM (n=54)	
	DR (n=87)	SR (n=54)	DR (n=32)	SR (n=32)	DR (n=75)	SR (n=50)	DR (n=27)	SR (n=27)
Sex	.59		.61		. > .99		.78	
Male	55 (63.2)	31 (57.4)	21 (65.6)	18 (56.3)	46 (61.3)	31 (62.0)	18 (66.7)	16 (59.3)
Female	32 (36.8)	23 (42.6)	11 (34.4)	14 (43.8)	29 (38.7)	19 (38.0)	9 (33.3)	11 (40.7)
Age at diagnosis, y	.23		.76		.10		.74	
< 50	18 (20.7)	16 (29.6)	6 (18.8)	8 (25.0)	16 (21.3)	18 (36.0)	5 (18.5)	7 (25.9)
≥50	69 (79.3)	38 (70.4)	26 (81.2)	24 (75.0)	59 (78.7)	32 (64.0)	22 (81.5)	20 (74.1)
Tumor sidedness	< .001		> .99		< .001		> .99	
Left sidedness	74 (85.1)	19 (35.2)	19 (59.4)	19 (59.4)	64 (85.3)	16 (32.0)	16 (59.3)	16 (59.3)
Right sidedness	13 (14.9)	35 (64.8)	13 (40.6)	13 (40.6)	11 (14.7)	34 (68.0)	11 (40.7)	11 (40.7)
Perioperative chemotherapy	.84		.79		> .99		.54	
No	65 (74.7)	42 (77.8)	23 (71.8)	21 (65.6)	56 (74.7)	37 (74.0)	21 (77.8)	18 (66.7)
Yes	22 (25.3)	12 (22.2)	9 (28.2)	11 (34.4)	19 (25.3)	13 (26.0)	6 (22.2)	9 (33.3)
c/p T stage	> .99		> .99		> .99		> .99	
T1-T2	11 (12.6)	6 (11.1)	4 (12.5)	3 (9.4)	8 (10.7)	6 (12.0)	3 (11.1)	2 (7.4)
T3-T4	76 (87.4)	48 (88.9)	28 (87.5)	29 (90.6)	67 (89.3)	44 (88.0)	24 (88.9)	25 (92.6)
c/p N stage	.78		.67		.89		.66	
N0	28 (32.2)	18 (33.3)	12 (37.5)	11 (34.4)	25 (33.3)	15 (30.0)	10 (37.0)	11 (40.7)
N1	37 (42.5)	20 (37.1)	14 (43.8)	12 (37.5)	30 (40.0)	20 (40.0)	10 (37.0)	7 (26.0)

© 2022 Wu Y et al. *JAMA Network Open.*

N2	22 (25.3)	16 (29.6)	6 (18.8)	9 (28.1)	20 (26.7)	15 (30.0)	7 (25.9)	9 (33.3)
Tumor differentiation	.71		＞.99		＞.99		.74	
Poor	29 (33.3)	16 (29.6)	9 (28.1)	10 (31.3)	22 (29.3)	15 (30.0)	5 (18.5)	7 (25.9)
Moderate/well	58 (66.7)	38 (70.4)	23 (71.9)	22 (68.7)	53 (70.7)	35 (70.0)	22 (81.5)	20 (74.1)
Metastatic sites								
Maximum size	.55		＞.99		.67		＞.99	
≤5cm	65 (74.7)	43 (79.6)	26 (81.3)	25 (78.1)	57 (76.0)	40 (80.0)	20 (74.1)	20 (74.1)
＞5cm	22 (25.3)	11 (20.4)	6 (18.7)	7 (21.9)	18 (24.0)	10 (20.0)	7 (25.9)	7 (25.9)
Numbers	＞.99		＞.99		.56		＞.99	
≤10	78 (89.7)	48 (88.9)	31 (96.9)	30 (93.8)	68 (90.7)	43 (86.0)	25 (92.6)	26 (96.3)
＞10	9 (10.3)	6 (11.1)	1 (3.1)	2 (6.2)	7 (9.3)	7 (14.0)	2 (7.4)	1 (3.7)
Distributions	＞.99		.77		＞.99		＞.99	
Unilobar	55 (63.2)	35 (64.8)	25 (78.1)	23 (71.9)	50 (66.7)	33 (66.0)	20 (74.1)	19 (70.4)
Bilobar	32 (36.8)	19 (35.2)	7 (21.9)	9 (28.1)	25 (33.3)	17 (34.0)	7 (25.9)	8 (29.6)
Anticipated hepatectomy	.69		＞.99		.67		＞.99	
Minor hepatectomy	64 (73.5)	42 (77.6)	25 (78.5)	26 (79.9)	57 (76.2)	40 (80.3)	21 (78.4)	21 (78.4)
Major hepatectomy [b]	23 (26.5)	12 (22.4)	7 (21.5)	6 (20.1)	18 (23.8)	10 (29.7)	6 (21.6)	6 (21.6)

Abbreviations: MT, mutated-type; OS, overall survival; CSS, cancer-specific survival; PSM, propensity score matching; DR, delayed resection; SR, simultaneous resection; y, years; c/p, clinically and pathologically; T, tumor; N, nodes.

a Categorical variables were presented as No. (%).

b Major hepatectomy is defined as resection of three or more liver segments.

© 2022 Wu Y et al. *JAMA Network Open.*

eTable 8. Univariate and Multivariate Analyses in Overall Survival and Cancer-Specific Survival in G12V *KRAS*-MT Patients With PSM

	**Overall survival**		**Cancer-specific survival**	
	**Univariate**	**Multivariate**	**Univariate**	**Multivariate**
**Characteristic**	***P* value**	**HR (95% CI), *P***	***P* value**	**HR (95% CI), *P***
Treatment	.38	NA	.20	NA
SR				
DR				
Sex	.20	NA	.25	NA
Male				
Female				
Perioperative chemotherapy	.06	NA	.06	NA
Yes				
No				
Tumor sidedness	.84	NA	.79	NA
Left sidedness				
Right sidedness				
c/p T stage	.94	NA	.55	NA
T1-2				
T3-4				
c/p N stage	.14	NA	.11	NA
N0				
N1				
N2				
Tumor differentiation	.32	NA	.82	NA
Moderate/well				
Poor				
Metastatic sites				
Maximum size	.57	NA	.94	NA
≤5cm				
＞5cm				
Numbers	.56	NA	.25	NA
≤10				
＞10				
Distributions	.74	NA	.23	NA
Unilobar				
Bilobar				
Anticipated hepatectomy	.46	NA	.36	NA
Minor hepatectomy				
Major hepatectomy [a]				

© 2022 Wu Y et al. *JAMA Network Open.*

Abbreviations: MT, mutated-type; PSM, propensity score matching; HR, hazard rate; CI, confidence interval; SR, simultaneous resection; DR, delayed resection; NA, not available; c/p, clinically and pathologically; T, tumor; N, nodes

© 2022 Wu Y et al. *JAMA Network Open.*
